# The Effect of Circumscribed Exposure to the Pan-Aurora Kinase Inhibitor VX-680 on Proliferating Euploid Cells

**DOI:** 10.3390/ijms232012104

**Published:** 2022-10-11

**Authors:** Xumei Liu, Qiong Shi, Namrta Choudhry, Ting Zhang, Hong Liu, Shenqiu Zhang, Jing Zhang, Dun Yang

**Affiliations:** 1 Hospital of Chengdu University of Traditional Chinese Medicine, Chengdu 610075, China; 2 Chengdu Anticancer Bioscience and J. Michael Bishop Institute of Cancer Research, Chengdu 610000, China; 3 Anticancer Bioscience (U.S.), San Francisco, CA 94080, USA; 4 Anticancer Bioscience (U.K.), St Andrews KY16 9QD, UK

**Keywords:** aurora kinase B, euploid cells, proliferative arrest, tetraploidy, senescence, VX-680, p53, Rb

## Abstract

Small molecule inhibitors of aurora kinases are currently being investigated in oncology clinical trials. The long-term effects of these inhibitors on proliferating euploid cells have not been adequately studied. We examined the effect of the reversible pan-aurora kinase inhibitor VX-680 on p53-competent human euploid cells. Circumscribed treatment with VX-680 blocked cytokinesis and arrested cells in G1 or a G1-like status. Approximately 70% of proliferatively arrested cells had 4N DNA content and abnormal nuclei. The remaining 30% of cells possessed 2N DNA content and normal nuclei. The proliferative arrest was not due to the activation of the tumor suppressor Rb and was instead associated with rapid induction of the p53–p21 pathway and p16. The induction was particularly evident in cells with nuclear abnormalities but was independent of activation of the DNA damage response. All of these effects were correlated with the potent inhibition of aurora kinase B. After release from VX-680, the cells with normal nuclei robustly resumed proliferation whereas the cells with abnormal nuclei underwent senescence. Irrespective of their nuclear morphology or DNA content, cells pre-treated with VX-680 failed to grow in soft agar or form tumors in mice. Our findings indicate that an intermittent treatment strategy might minimize the on-target side effects of Aurora Kinase B (AURKB) inhibitory therapies. The strategy allows a significant fraction of dividing normal cells to resume proliferation.

## 1. Introduction

Aurora kinases (AURKs) have been intensely studied as anticancer targets, and their small molecule inhibitors have entered various phases of clinical trials [1,2]. The first-in-class AURK inhibitor VX-680/MK-0457/Tozasertib invariably works to obstruct the proliferation of cancer cell lines, trigger apoptosis, and elicit a broad therapeutic effect at low nanomolar potency in mouse tumor models [3]. Intriguingly, VX-680 selectively kills cells overexpressing a homolog of the avian viral myelocytomatosis oncoprotein (Myc) by the inhibition of aurora kinase B (AURKB) [4]. AURKB is the catalytic subunit of the chromosomal passenger protein (CPP) complex, which acts as a master coordinator of karyokinesis and cytokinesis [5]. Overexpressed Myc primes cells to the lethal effect of VX-680 by promoting illegal DNA synthesis and mitosis in the absence of cytokinesis [4,6]. This Myc activity allows pulsed treatments with VX-680 to achieve an enduring anticancer effect by eliciting delayed lethality without the requirement of continuous exposure to the drug.

Pulsed treatments with VX-680 might also minimize its toxicity for dividing normal cells because euploid cells readily resume proliferation after release from treatment with VX-680 [4]. Upon inhibition of AURKB, proliferating normal cells suffer a cytokinetic failure, and the resulting tetraploid cells are well known to undergo senescence [7,8,9]. Therefore, the rapid renewal of proliferation after the withdrawal of VX-680 is intriguing. The mechanism underpinning the phenomenon needs to be defined.

In addition, whether the inhibition of AURKB could accidentally promote tumorigenesis warrants careful investigation because tetraploidization could elicit opposite effects on tumorigenesis in a context-dependent manner. On the one hand, tetraploid cells represent an intermediary state in the neoplastic transformation linking euploidy and aneuploidy [10,11]. This tumor-promoting property stems from the chromosome instability intrinsic to tetraploid cells, which also have a higher tolerance to the loss of chromosomes than diploid cells [10,11]. Conversely, tetraploidy could suppress tumorigenesis [12,13], which is consistent with the findings that treatment-induced tetraploid cells frequently undergo a form of terminal arrest known as senescence [7]. It is still under debate whether a tetraploidy checkpoint operates in mammalian cells. Nonetheless, halting DNA synthesis in tetraploid cells that have been induced by antimitotic agents is typically associated with the induction of the p53 tumor suppressor protein and its downstream target p21, an inhibitor of cyclin-dependent kinases (Cdks) [14,15]. Besides p53, the tumor suppressor protein retinoblastoma (Rb) also plays a role in preventing treatment-induced polyploid cells from synthesizing DNA illegitimately [16,17]. These observations support the conventional views of both senescence and polyploidy as not only intrinsic tumor-suppressing mechanisms but also alternative therapeutic endpoints [18].

It is also possible that senescence may eventually facilitate tumor relapse. Defying the otherwise irreversible senescent cell arrest, rare once senescent cancer cells can eventually renew proliferation [19]. Some senescent cancer cells may even maintain their long-term viability by feeding on surrounding cells [20,21]. Furthermore, senescent cells often secrete diverse soluble factors, which can impact the tumor microenvironment and/or local immune response. The combined consequences of these properties could either promote or suppress tumorigenesis [13,22].

Here, we investigated the long-term effect of VX-680 on p53-competent human immortalized euploid retinal pigment epithelial cells (RPE) and human primary fibroblasts (IMR-90 and MRC-5). We addressed the hypothesis that the circumscribed application of an AURK inhibitor would lead to the recovery of proliferation and whether such recovery would have an unwanted tumorigenic effect. VX-680 elicited a G1-like arrest of cells with either 2N or 4N DNA content. A small fraction of proliferatively arrested cells did not suffer a cytokinetic failure in the presence of VX-680 and robustly resumed proliferation after release from treatment. In contrast, the majority of cells developed abnormal nuclei when exposed to VX-680. The cells with abnormal nuclear morphology underwent a permanent arrest of proliferation and developed various phenotypes characteristic of senescence even after discontinuation of the treatment. The circumscribed exposure of cells to VX-680 failed to transform them in vitro or engender tumorigenesis in xenograft experiments. These data provide new insight into the mechanism underpinning the rapid repopulation of euploid cells after treatment with VX-680. They also indicate that tetraploid or senescent cells resulting from VX-680 treatment of p53-competent euploid cells are not necessarily tumorigenic.

## 2. Results

### 2.1. Euploid Cells Are Arrested in G1 by VX-680 Independent of the Tumor Suppressor Rb

**Figure 1:**
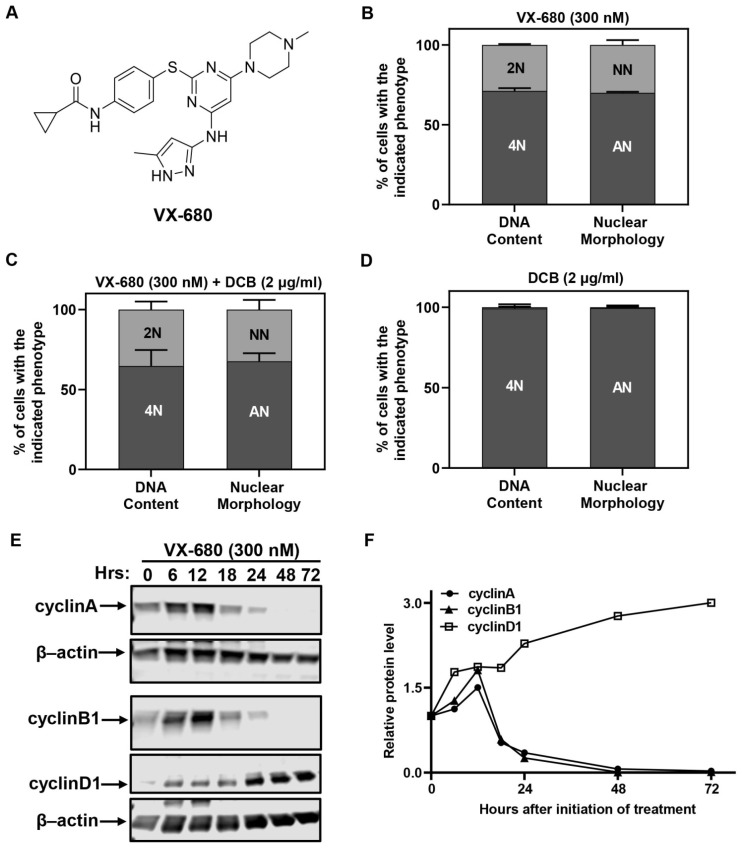
VX-680 arrests euploid cells in G1 with either 2N or 4N DNA content. (**A**) Chemical structure of VX-680. (**B**–**D**) RPE-NEO cells were treated with VX-680 (**B**), VX-680 + DCB (**C**) or DCB (**D**) for 3 days and then analyzed for DNA content by flow cytometry after propidium iodide (PI) staining or nuclear morphology after 4′,6-diamidino-2-phenylindole (DAPI) staining. (**E**,**F**) Western analysis (**E**) was used to quantify (**F**) the expression of cell cycle regulatory proteins with time of exposure. The blots were cropped, and the full-length original blots are presented in Supplementary Figure S4A,B. NN, cells with a normal nucleus; AN, cells with an abnormal nucleus. Data presented in (**B**–**D**) are expressed as the mean ± SD from three independent experiments, conducted in triplicate (*n* = 9). The quantification of a representative experiment in (**E**) is presented in (**F**). Western blot was performed in two independent experiments. The quantification of a representative experiment in E has been presented in F. Two independent experiments produced the same trend.

We previously demonstrated a synthetic lethal interaction between overexpressed Myc and VX-680 in a pair of model cell lines RPE-NEO and RPE-MYC [4]. In response to VX-680, control RPE-NEO cells underwent proliferative arrest as opposed to cell death. The cells could resume robust proliferation after the treatment was withdrawn. To further characterize the proliferative arrest of RPE-NEO cells by VX-680 (Figure 1A), we utilized the treatment scheme developed in our previous study [4]. Cells were exposed to 300 nM of VX-680 for three days, which is 2-fold higher than its EC_50_, and then the drug was removed. This drug concentration represents the threshold effective concentration that achieves complete inhibition of AURKB in RPE-NEO cells (Supplementary Figure S1). A 50% inhibition of Aurora Kinase A (AURKA) was observed at 12 but not 24–48 h after the initiation of the treatment. Treatment for three days allowed VX-680 to kill nearly all RPE-MYC cells after withdrawal of the treatment [4]. This treatment scheme also allowed the RPE-NEO cells to resume robust proliferation after the withdrawal of VX-680 [4].

We analyzed the DNA content by flow cytometry and nuclear morphology by microscopy at the end of the 3-day treatment. Each assay identified two distinct fractions of cells. About 70% of cells had 4N DNA content or a visually abnormal nucleus (Figure 1B), denoted by either a bilobular nucleus or a large giant nucleus. This phenotype has been described before and has been attributed to cytokinetic failure [4]. We referred to the cells with abnormal nuclei as AN cells in this study. Surprisingly, the remaining 30% of cells had 2N DNA content or a normal nucleus and were referred to as NN cells (Figure 1B). We used dihydrocytochalasin B (DCB), which inhibits the completion of cytokinesis, to ascertain that NN cells did not enter DNA synthesis in the presence of VX-680. DCB was used at a concentration of 2 µg/mL [23], the threshold effective concentration that blocks cytokinesis with 100% penetrance. Approximately 30% of NN cells or 2N cells appeared when RPE-NEO cells were subjected to a combination treatment with VX-680 and DCB (Figure 1C). In contrast, all cells became tetraploid and had two well-separated nuclei when treated with DCB alone (Figure 1D). Collectively, these data implied that two distinct subpopulations arose in response to VX-680. Proliferative arrest by VX-680 was independent of the nuclear morphology and DNA content. The findings from the DCB trapping experiment imply that NN cells never progressed into the S phase in the presence of VX-680.

A Western blot analysis of cell cycle-associated cyclins was carried out in cells treated with VX-680. The abundance of G1-type cyclin D1 increased at the expense of mitotic cyclins A and B1 (Figure 1E,F). Before declining and eventually disappearing, the abundance of both cyclin A and B1 transiently increased until 12 h after initiation of treatment. The increase likely reflected a transient arrest of cells at the G2/M transition due to inhibition of AURKA, which is reported to promote the G2/M transition [24].

Together, these data suggest that RPE-NEO cells were arrested in G1 or a G1-like state, irrespective of their DNA content. The arrest was not simply the consequence of a prior cytokinetic failure because a fraction of cells never progressed into cytokinesis. All RPE-MYC cells could undergo multiple runs of DNA replication when VX-680 alone was used to block cytokinesis [4]. Additionally, we can conclude that overexpression of Myc works to overcome this treatment-induced cell cycle arrest, forcing more cells to undergo a defective mitosis and increasing their susceptibility to apoptosis or polyploidy.

**Figure 2:**
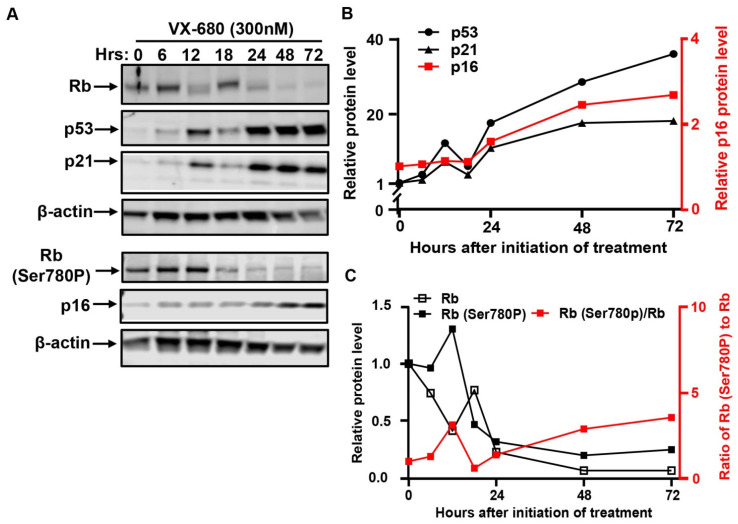
VX-680 activates the p53-mediated checkpoint pathway but not the tumor suppressor Rb. RPE-NEO cells were exposed to 300 nM of VX-680 for three days. Cells were collected at the indicated time points after the initiation of drug treatment for the analysis of the indicated proteins by Western blot. The blots were cropped, and the full-length original blots are presented in Supplementary Figure S4B,C. Quantification of p53, p21, and p16 in (**A**) is presented in (**B**). Quantification of total Rb, Rb phosphorylated at Ser780, and the percentage of Rb phosphorylation at Ser780 is shown in (**C**). A representative experiment is shown. Western blot was performed in two independent experiments. The quantification of a representative experiment in A has been presented in B and C. Two independent experiments produced the same trend.

The dephosphorylation of the tumor suppressor retinoblastoma (Rb) at Ser780 is critical for the G1/S transition [16]. AURKB has been implicated in the inactivation of the tumor suppressor by phosphorylation of Rb at Ser780 [25]. We observed that phosphorylated Rb at Ser780 declined in response to VX-680 (Figure 2A,C). However, the reduction occurred concurrently with a decline in total Rb protein. In particular, Rb protein vanished after 24 h following the initiation of treatment. Thus, the proliferative arrest was unlikely to be solely due to the activation of Rb despite AURKB inhibition by VX-680.

### 2.2. VX-680 Activates the p53-Dependent G1 Checkpoint without Evoking the DNA Damage Response

**Figure 3:**
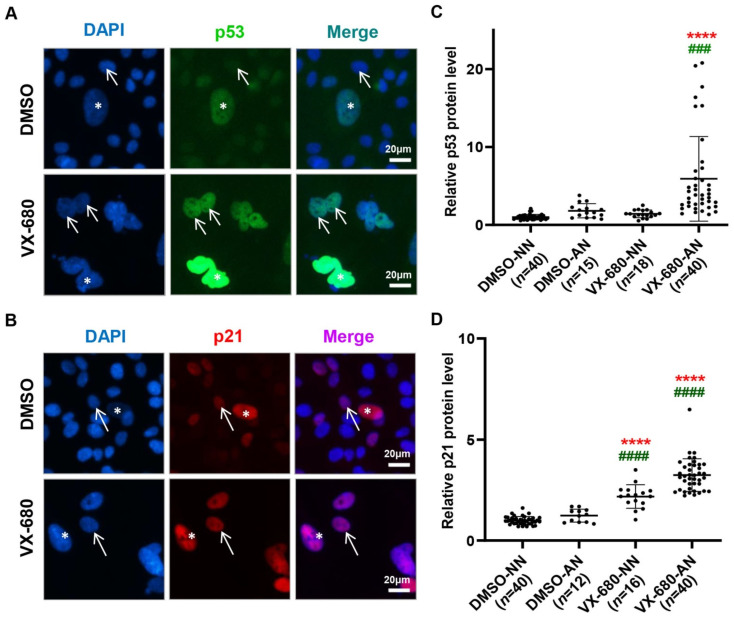
VX-680 induces p53 in AN cells but elevates p21 in both NN and AN cells. RPE-NEO cells were exposed to 300 nM of VX-680 for three days before immunofluorescent analysis for p53 (**A**) and p21 (**B**). The arrows in (**A**,**B**) denote cells with a normal nucleus (NN) and the stars point to cells with an abnormal nucleus (AN). Rare cells with AN exist naturally in the absence of any treatment. The immunofluorescence intensity of p53 (**C**) and p21 (**D**) was quantified in cells with either an NN or AN separately. The rare cells with an abnormal nucleus in the DMSO group were also analyzed. The data in (**C**,**D**) were values obtained from one representative independent experiment with a triplicate and were normalized to the average value of the cells with an NN in the control DMSO group. The *p* values were calculated with the Dunnett test following one-way ANOVA. ****, *p* < 0.0001 when compared with the DMSO-NN group. ###, *p* <0.001 and ####, *p* < 0.0001 when compared with the DMSO-AN group. Black dots in panels C and D indicate the number of replicates used to quantify, p53 and p21 levels respectively. Replicate numbers (*n*) are given in brackets on the x-axis.

The induction of the p53–p21-dependent checkpoint pathway mediates proliferative arrest in response to diverse forms of cellular stress [26]. p53 and its target gene p21 were concurrently induced by VX-680 (Figure 2A,B), as was documented previously [4]. The induction of both proteins became appreciable after 6 h of treatment and further increased from 24 to 72 h. Since two discrete fractions of cells arose in response to VX-680, AN, and NN cells, we performed immunofluorescence analysis to understand if both fractions of cells had responded through activation of the p53–p21 pathway. The abundance of p53 protein robustly increased in AN cells, but not NN cells (Figure 3A,C). This differential induction suggests that VX-680 exposure alone is not sufficient to induce p53 in all cells. In the absence of VX-680, NN cells and spontaneously formed AN cells had comparable, low p53 abundance, indicating that cytokinetic failure itself was not sufficient to induce p53 (Figure 3C). Published studies have implicated AURKA and AURKB in destabilizing p53 through phosphorylation [27,28]. However, the selective accumulation of p53 in AN but not NN cells implies that the induction could not be accredited to the loss of AURK-mediated p53 phosphorylation, as AURK inhibition occurs in both cell populations. Instead, it appears that additional cellular defects or deficiencies lower the threshold for p53 activation in VX-680-treated AN cells. We suggest that such a deficiency must arise though combined AURK inhibition and cytokinesis blockade, as neither AURKB inhibition nor polyploidy alone resulted in efficient p53 activation.

Observations regarding the induction of p21^WAF1^ following VX-680 support the notion that additional pathways may work with p53 induction to effect arrest. Distinct from p53, the induction of p21 by VX-680 also occurred, albeit to a lesser extent, in NN cells (Figure 3B,D). Since p53 was not appreciably elevated, the mechanism underlying the induction of p21 was unclear. Consistent with p53 induction in AN cells by VX-680, p21 was elevated to a level higher than that seen in NN cells treated with VX-680 (Figure 3D). Thus, AN cells might harbor a more permissive cellular context than NN cells, allowing more robust p53 induction by VX-680.

We excluded the possibility that the induction of the p53–p21 pathway was due to DNA damage (Supplementary Figure S2). H2A histone family member X (H2A.X) is phosphorylated at Ser139 in response to double-stranded DNA damage and plays an essential role in the G2/M checkpoint-mediated cell cycle arrest and DNA repair [29]. Likewise, the phosphorylation of p53 at Ser15 occurs in response to DNA damage and this can facilitate its stabilization [30]. Neither p53 nor H2A.X displayed detectable levels of phosphorylation at these sites in cells treated with VX-680 (Supplementary Figure S2A). Cells showed robust phosphorylation in response to the DNA damage agent Doxorubicin (Supplementary Figure S2B), validating the specificity of these two antibodies.

To further explore the mechanism underpinning p53 induction by VX-680, we examined the oncogenic transcription factors yes-associated protein/tafazzin (YAP/TAZ), which act as the effector of the Hippo tumor suppressor pathway [31]. The inactivation of the Hippo tumor suppressor signaling pathway promotes the proliferation and suppresses the expression of p53. Surprisingly, nuclear YAP/TAZ proteins were moderately upregulated, rather than suppressed, in cells treated with VX-680 (Supplementary Figure S3). This observation was true for cells with a normal or abnormal nucleus. This finding implies that neither proliferative arrest nor p53 induction could be ascribed to activation of the Hippo signaling pathway, which decreases the nuclear levels of YAP/TAZ [31].

We conclude that the proliferative arrest by VX-680 had characteristics of the activation of the p53-dependent checkpoint response. Upon exposure to VX-680, a fraction of cells were arrested in G1 with 2N DNA content. This fraction of cells did not undergo cytokinesis in the presence of VX-680. The other cells had 4N DNA content with abnormal nuclei and were arrested in a G1-like state. Consistent with a published study [25], our findings also implicated AURKB in promoting the G/S transition. However, distinct from the report, we found that the disablement of AURKB with VX-680 elicited a G1 arrest that may not be mediated primarily through Rb, but by p53 induction.

### 2.3. The Arrest of DNA Synthesis in NN Cells Is Reversible

**Figure 4:**
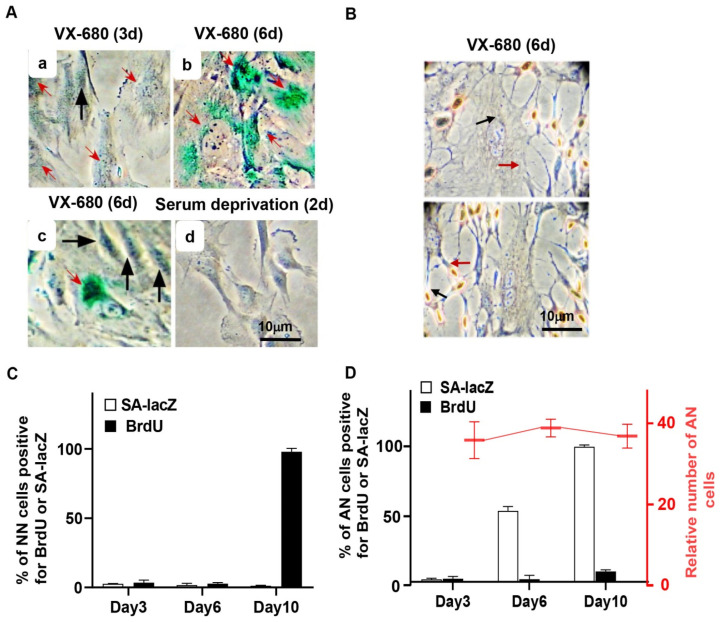
The short-term inhibition of AURKB elicits a reversible arrest of proliferation in NN cells but senescence in AN cells. RPE-NEO cells were treated with 300 nM of VX-680 for 3 days and then transferred to the fresh medium in the absence of the drug. (**A**) Assays for senescence-associated β-galactosidase (SA-lacZ) activity were performed at 3 (**a**) or 6 days (**b**,**c**) after the administration of the drug. This assay was also performed with RPE-NEO cells that were proliferatively arrested by deprivation of serum for 2 days (**d**). Black arrows point to cells with normal nuclei, and red arrows denote cells with abnormal nuclei. (**B**) Two independent images of cells assayed for proliferation with the BrdU incorporation assay on day 6 of VX-680 treatment. (**C**,**D**) The percentage of cells positive for the indicated assays. RPE-NEO cells were treated with 300 nM of VX-680 for 3 days and then transferred to the fresh medium in the absence of the drug. Assays for SA-lacZ activity (□) and BrdU incorporation (■) were performed at various lengths of time after the administration of VX-680. Quantification of cells positive for these assays was presented in (**C**) for the cells with normal nuclei and in (**D**) for cells with abnormal nuclei. Approximately 200 AN or NN cells were scored per experiment for each time point in each group. AN cells were also enumerated under a tissue culture microscope in 20 randomly chosen fields, and the average AN number in each field at each time point is presented as the relative cell number in (**D**). All data presented are the mean ± SD from three independent experiments with counts done in triplicate (*n* = 9).

RPE-NEO cells could vigorously renew proliferation after release from treatment with VX-680 [4]. We investigated whether both the NN and AN fractions reentered the cell cycle. The 5′-Bromo-2″-deoxyuridine (BrdU) incorporation assay, which characteristically stains cells in the S phase [32], was used to mark proliferative cells. All NN cells were BrdU^+^ after ten days of administration of VX-680 (Figure 4B,C). In contrast, AN cells were negative (Figure 4B,D). The AN population of cells was readily distinguishable from the surrounding NN cells under an inverted tissue culture microscope, so this population could be quantified by simply counting this morphologically distinct subpopulation. Consistent with an absence of DNA synthesis, the number of AN cells remained constant during the assay despite the fact that cell density increased and the plate reached confluence (Figure 4D). Collectively, these data demonstrate that the population increase after release from VX-680 treatment reflects the renewed proliferation of NN cells rather than AN cells.

### 2.4. The Arrest of the DNA Synthesis in AN Cells Is Associated with Senescence Phenotypes

After release from VX-680 treatment, AN cells gradually became enlarged and flattened, a morphological feature characteristic of senescent cells. This observation prompted us to examine senescence-associated β-galactosidase (SA-LacZ) activity and p16^INK4A^, a CDK inhibitor involved in executing senescence [33]. We detected SA-LacZ activity in 0%, 50%, and 100% of the AN cells on days 3, 6, and 10, respectively, after initiation of VX-680 treatment (Figure 4A,D). As expected, NN cells were negative for SA-LacZ staining (Figure 4A,C). As a negative control, we stained cells proliferatively arrested in the absence of serum. None of the serum-starved cells tested positive for SA-LacZ activity (Figure 4A). Consistent with the SA-LacZ staining of AN cells and mutual induction of p16 during cellular senescence in response to various stresses [33], p16 was robustly elevated subsequent to the p53 and p21 accumulation in response to VX-680 (Figure 2A,B).

**Figure 5:**
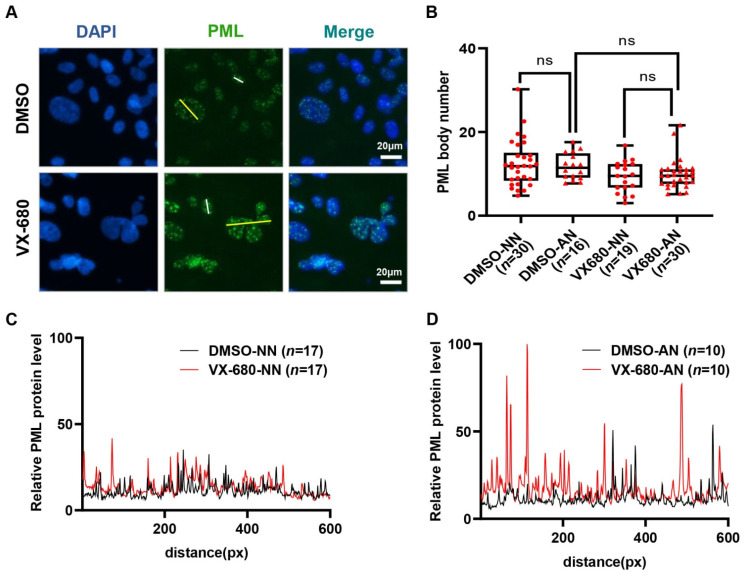
Treatment with VX-680 increases the immunofluorescent staining intensity of PML bodies in cells with an abnormal nucleus. (**A**) RPE-NEO cells were treated with either DMSO or 300 nM of VX-680 for 3 days before being fixed for the immunofluorescence analysis of PML. (**B**) The number of PML bodies in cells with either a normal nucleus (NN) or an abnormal nucleus (AN) was quantified separately for both the DMSO and VX-680 groups. The relative number of PML bodies after normalization to the nuclear sizes is presented. (**C**,**D**) The intensity of the PML immunofluorescence in NN (**C**) or AN (**D**) cells in the DMSO group and VX-680 group was quantified in randomly chosen cells. Representative data are shown from one of three independent experiments in (**C**,**D**). The analyzed path for PML signal measurement was along the white lines (NN) and yellow lines (AN) indicated in (**A**). The significance of the data in (**B**) was calculated with the Tukey test following one-way ANOVA (ns is not significant). Red dots (NN) and triangles (AN) in panel B indicate the number of replicates used to analysis of PML bodies. Replicate numbers (*n*) are given in brackets on the x-axis.

The overexpression of promyelocytic leukemia protein (PML) has also been implicated in p53-mediated senescence [34,35]. Consistently, in SA-lacZ-positive AN cells, the intensity of PML bodies increased greatly compared with NN cells in the VX-680 treatment group (Figure 5A,C,D). Rare binucleated cells existed in the control group, presumably resulting from a spontaneous cytokinetic failure. These binucleated cells had an intensity of PML body staining similar to that of 2n cells with a normal nucleus (Figure 5A). Thus, the increase in PML staining intensity, presumably a function of PML protein accumulation in the PML body, could not be accredited solely to either tetraploidy or abnormal nuclear morphology. Since VX-680 treatment enhanced the intensity of PML bodies selectively in AN but not in NN cells (Figure 5C,D), we concluded that neither VX-680 alone nor cytokinetic failure alone is sufficient to induce PML accumulation in PML bodies, a finding similar to the induction of p53 and accumulation of SA-laZ staining. We also enumerated the PML bodies, not just quantifying the intensity of staining. The number of PML bodies increased more than 2-fold in the AN cells compared with the NN cells. However, after normalization to nuclear size, the number of PML bodies was comparable between AN and NN cells (Figure 5B). Therefore, the PML content, not the number of PML bodies was increased in VX-680 treated cells that experienced a cytokinetic failure.

We conclude that brief exposure to VX-680 elicited an irreversible arrest of DNA synthesis and induction of senescence, only in AN cells of the population. The same treatment reversibly arrested proliferation in a minor fraction of cells, the NN population. These data contrast with the finding that cells-overexpressing Myc underwent multiple rounds of DNA synthesis before a delayed death after release from treatment with VX-680 [4]. This contrast implies that pulsed administration of VX-680 might not only achieve sustained therapeutic efficacy, as we suggested previously [4], but also minimize toxicity for proliferating normal cells by allowing a fraction of the normal cell population to renew proliferation.

### 2.5. The p53-Competent Euploid RPE Cells Are not Tumorigenic after Release from VX-680 Treatment

**Figure 6:**
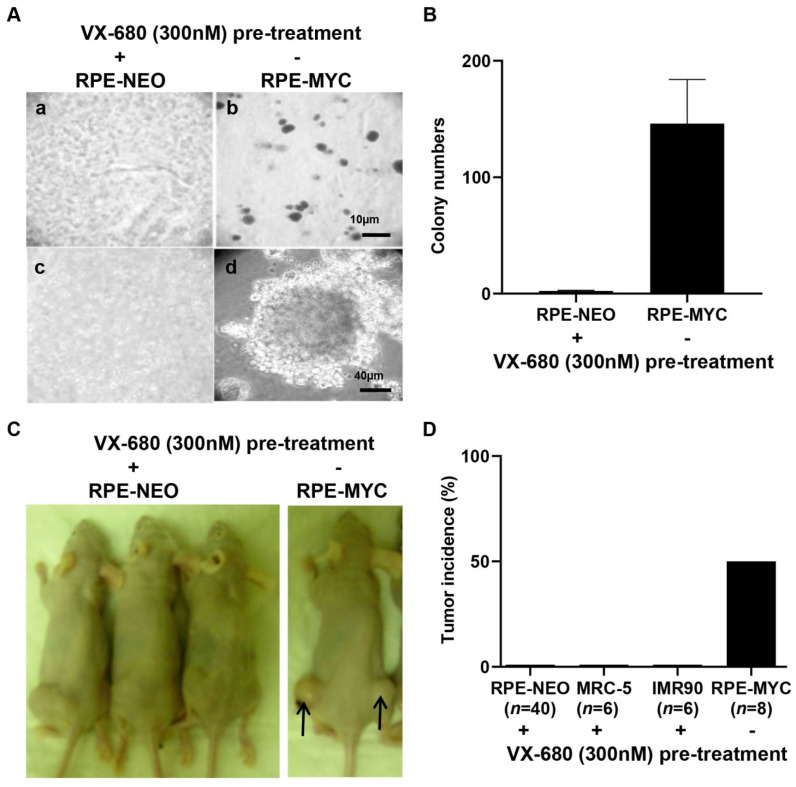
The tetraploid cells induced by a brief treatment of euploid cells with VX-680 are not tumorigenic. RPE-NEO cells were treated with 300 nM of VX-680 for 3 days to induce tetraploidy in about 70% of the population before being analyzed for tumorigenic potential. Untreated RPE-MYC cells were used as a positive control. (**A**) Ten thousand RPE-NEO cells pretreated with VX-680 were assayed for anchorage-independent growth in soft agar (**a**,**c**). The same number of untreated RPE-MYC cells was used in (**b**) and (**d**). The scale bar in b indicates 10 µm, whereas that in d is 40 µm. (**B**) The colonies in 20 randomly chosen fields were quantified under a tissue culture microscope at a magnification of ×200. (**C**,**D**) The indicated cells pretreated with VX-680 and untreated RPE-MYC cells were assayed for tumor formation in immunocompromised female Nu/Nu mice. Both flanks of each mouse were implanted with one million cells per flank. n indicates the number of injection sites. Representative images are presented in (**C**). The percentage of the injection sites that formed tumors is presented in (**D**). Note the lack of clear cell, distinguishable clusters in (**A**) and (**C**) compared to (**B**) and (**D**), as these images are of single cells or small clusters within a three-dimensional background of agarose matrix.

Both tetraploidy and senescence have been implicated in facilitating tumorigenesis [12,13]. Therefore, we investigated whether short-term treatment with VX-680 could transform euploid cells in vitro and render them tumorigenic in vivo. For this purpose, the soft agar assay was performed, as it is known to be the most stringent test for malignant transformation in vitro. RPE-NEO cells failed to grow in soft agar irrespective of treatment with VX-680. Untreated RPE-MYC cells, a positive control for the transformation assay, formed hundreds of colonies (Figure 6A,B). Next, a xenograft assay was conducted with RPE-NEO cells pre-treated with VX-680. One million live RPE-NEO cells after pre-treatment with VX-680 failed to develop tumors even six months post-implantation into immunocompromised nude mice (Figure 6C,D). This failure was unlikely due to the rejection of the xenograft by the residual immune function in nude mice because untreated RPE-MYC cells formed tumors within two months with a penetrance of 50% (Figure 6C,D). We extended this study to human primary fibroblasts, IMR-90 and MRC-5. Both cell lines failed to produce tumors after similar pre-treatment with VX-680 (Figure 6D). We concluded from these studies that VX-680 is not transforming for normal cells.

## 3. Discussion

### 3.1. The G1 Arrest of p53-Competent Euploid Cells Elicited by VX-680

Disablement of AURKB with VX-680 led to a blockage of both the G1/S transition and cytokinesis, engendering a mixed population of the proliferatively arrested NN cells and AN cells. NN cells were arrested with 2N DNA content without a prior round of cytokinesis. The AN cells were arrested cells but with 4N DNA content, presumably having suffered a cytokinetic failure. Therefore, NN cells and AN cells underwent transient proliferative arrest and senescence, respectively.

Overexpression of Myc overcomes the G1 arrest but not cytokinetic obstruction in cells exposed to VX-680 [4]. Consequently, polyploid cells developed in this study, consistent with the formation of polyploid RPE-MYC cells following VX-680 treatment that eventually succumb to cell death, even in the absence of continued treatment [4]. These findings support that pulsed treatment with VX-680 might mitigate its toxicity for proliferating normal tissues by allowing the preservation of tissue homeostasis. Meanwhile, pulsed administration might achieve long-lasting therapeutic efficacy against Myc tumors by eliciting delayed toxicity.

Pulse treatment has been typically utilized to reduce the toxicity of chemotherapies. This treatment strategy is believed to sustain tissue homeostasis by allowing the proliferation of stem cells to replace dividing normal cells killed by the pulse treatment. We provide another perspective that pulse treatment with certain antimitotic agents such as VX-680 might be tailored to reduce toxicity by allowing the renewed proliferation of dividing normal cells. Since our observations are limited by the two types of cells examined, it would be necessary to examine other cell types to understand whether our findings are generally applicable.

### 3.2. p53-Competent Euploid Cells Undergo Senescence, Not Transformation

Tetraploid cells contain twice the normal amount of DNA, and the polyploid state is believed to be an intermediate cell state that fuels the malignant progression of cancer cells by promoting aneuploidy [10]. In support of this hypothesis, tetraploid mouse mammary epithelial cells induced by transiently blocking cytokinesis could develop tumors in allograft experiments [36]. Later studies demonstrated that tetraploidization may promote tumorigenesis in some tissues whereas it may suppress tumorigenesis in other tissues [37,38]. These opposing effects make it difficult to forecast the tumorigenic potential of tetraploid cells. Here, we experimentally tested the tumorigenic potential of the tetraploidization of p53-competent RPE cells and fibroblasts induced by VX-680. We discovered that such engineered tetraploid cells failed to grow anchorage independently in soft agar assays and did not form tumors in immunocompromised mice. Instead, they underwent an irreversible proliferative arrest associated with multiple morphological and molecular characteristics of senescence. Under the same treatment regimen, some cells were arrested in G1 with 2N DNA content, and these cells did not show signs of senescence. This difference between NN and AN cells indicates that VX-680 does not necessarily induce senescence. Instead, VX-680 might synergize with cytokinetic failure, or secondary cellular abnormality resulting from cytokinetic failure, to trigger senescence.

### 3.3. VX-680 Preferentially Induces the p53–p21 Pathway in AN Cells Independent of DNA Damage

Cellular senescence is known to be associated with the induction of the p53–p21 pathway. We show that in AN cells, VX-680 disabled AURKB and activated this checkpoint pathway. The increase in p53 abundance in AN cells by VX-680 is consistent with the known role of AURKs in destabilizing p53 [27,28]. However, VX-680 is not sufficient to induce readily detectable levels of nuclear p53 in NN cells. VX-680 has been reported to trigger DNA damage, which, in turn, activates the p53–p21 pathway, in a human cancer cell line [39], but we found no evidence that VX-680 damaged DNA in euploid RPE cells. We note that antimitotic agents, such as actin inhibitors or spindle toxins, elicit a p53-dependent arrest of cells in a tetraploid state, despite the fact that none of these reagents can inhibit AURK catalytic activity or damage DNA [40,41]. Therefore, DNA damage, per se, may not be the route by which the p53–p21 pathways are activated in AN cells. Our studies also demonstrate that the induction of p53 in tetraploid cells is not triggered by properties such as the presence of supernumerary centrosomes, bi-nucleation, having 4N DNA content, or cytokinetic failure [42], as tetraploid cells formed without VX-680 treatment did not have the p53 activation. Thus, the disturbance of mitosis and cytokinesis alongside VX-680 might generate a cryptical signal that together trigger an upsurge of p53 and, consequently, the inhibition of DNA synthesis.

## 4. Materials and Methods

The purchase information is stated for all chemicals. All equipment and software used for the figures and statistical analysis are mentioned. All methods were performed under the relevant guidelines and regulations.

### 4.1. Cell Culture and Treatment

The generation of RPE-NEO and RPE-MYC cells was previously described [43]. Briefly, RPE-NEO and RPE-MYC cells were generated by transfecting hTERT-immortalized human primary retinal pigment epithelial cells (RPE-hTERT) with a vector expressing the Neomycin resistance gene (*Neo*) alone or alongside the *MYC* oncogene, respectively, followed by selection with 800 of μg/mL G418/Geneticin. The RPE series of isogenic cells were cultured in DMEM (Cat. No. 12100061) supplemented with 5% of fetal bovine serum (Cat. No.16140-063), penicillin (100 U/mL)-streptomycin (100 μg/mL)(Cat. No. 15140-122), 2 mM L-glutamine (200 mM solution, Cat. No. 25030081), and 1 mM sodium pyruvate (100 mM solution, Cat. No. 11360070) at 37 °C in a humidified incubator with 5% CO_2_. The reagent used in cell culture was sourced from Gibco Construction Cleveland, TN, USA. The identity of the cell lines was authenticated through the human STR profiling service of ATCC. Mycoplasma contamination was evaluated by PCR-based assays using a Universal Mycoplasma Detection Kit (ATCC^®^ 30–1012K™, Gaithersburg, MD, USA). Each cell line was then amplified and stored in aliquots. One aliquot was used only for 10–15 passages without additional verification. Small molecules were used at the following final concentrations: VX-680, 300 nM; DCB, 2 μg/mL. VX-680 was prepared as a 3000× stock solution in DMSO and the DCB as a 1000× stock solution. VX-680 was purchased from Kava Technology, San Diego, CA, USA, while DCB was obtained from Calbiochem, Sigma-Aldrich, St. Louis, MO, USA. The identity and purity of all chemicals were validated in-house by HPLC and LC-MS.

### 4.2. Western Blot Analysis

The immunoblotting procedure was described before [4]. RIPA buffer was used to lyse cells, supplemented with a cocktail of phosphatase (#P1082) and protease inhibitors (#P1005). Cell lysates were sonicated for 10 s and centrifuged at 12000× rpm for 15 min. Gel electrophoresis of proteins proceeded under the condition of 110 V for 2 h before the proteins were transferred onto a PVDF membrane at 60 V for 60 min. The membrane was then blocked with 5% of non-fat milk in TBST buffer for 1.5 h at room temperature. For the detection of the proteins of interest, the membrane was incubated with primary antibodies overnight at 4 ℃, washed with TBST three times for 10 min each, and probed with a secondary antibody. Primary antibodies against phospho-Rb at Ser780 (#9307), phospho-p53 at Ser15 (#9284), phospho-p53 at Ser20 (#9287), phospho-Histone H3 at Ser10 (#53348), and phospho-AURKA at Thr288 (#3079) were purchased from Cell Signaling Technology, Danvers, MA, USA. Antibodies against Cyclin A (sc-751), Cyclin B1 (sc-752), Cyclin D1 (sc-246), p53 (sc-126), p21 (sc-391), Rb (sc-50), and p16 (sc-759) were obtained from Santa Cruz Biotechnology, Dallas, TX, USA. Rabbit antibodies for Actin (20536-1-AP) and Histone-H3 (17168-1-AP) and mouse antibodies for Actin (66009-1-Ig) and AURKA (66757-1-Ig) were purchased from Proteintech. Rabbit antibody against H2A.X (phospho-Ser139) (#07-164) was obtained from Merck, NJ, USA. Secondary antibodies including IRDye^®^ 800CW anti-Mouse antibody (Lot#C91210-09) and IRDye^®^ 680RD anti-Rabbit antibody (Lot#D00115-06) were obtained from Jackson ImmunoResearch Laboratories, West Grove, PA, USA.

Images were obtained from the Odyssey CLx Imaging System and processed with Image Studio Ver 5.2. The imaging system and software are sourced from LI-COR, Lincoln, NE, USA. To determine relative protein densities, the signals for the indicated proteins at each time point were first normalized to the internal control signals of actin in the same lane and then further normalized to their respective signals at time zero, which were set as 1.0. The relative protein abundance was used to calculate the percentage of protein phosphorylation.

### 4.3. Immunofluorescence

Cells were cultured on glass slips in a 6-well plate and fixed with 4% paraformaldehyde for 10 min before being permeabilized with 0.5% Triton X-100 for 10 min. After being blocked with 5% BSA at room temperature for 1 h, cells were incubated with primary antibodies overnight at 4 °C. Antibodies against p53 (sc-126), p21 (sc-397), and PML (sc-966) were purchased from Santa Cruz Biotechnology, Dallas, TX, United States USA. The anti-YAP antibody (#4912) was from Cell Signaling Technology, Danvers, MA, USA. All primary antibodies were used at 1:50 dilution. The cells were then stained with two secondary antibodies, including an FITC-conjugated anti-mouse antibody (115-095-003) and a TRITC-conjugated anti-rabbit antibody (111-095-003), from Jackson ImmunoResearch Laboratories, West Grove, PA, USA, for 2 h at room temperature at 1:100 dilution before mounted in DAPI-containing Fluoromount-GTM (36308ES20, YEASEN), Shanghai, China. Images were acquired and processed using an EVOS FL Auto Cell Imaging System, Thermo Fisher, Waltham, MA, USA.

### 4.4. BrdU Cellular Proliferation Assay

As described [44], cells were exposed for 16 h to 10 µM BrdU and then fixed in 75% ethanol. The nuclear incorporation of BrdU was visualized by immunostaining with a cell proliferation kit (QIA58, EMD Millipore, Merck KGaA, Germany).

### 4.5. Senescence-Associated-β-Galactosidase Assay

Cells were seeded and cultured on fibronectin-treated glass coverslips. At the time of harvest, cells on coverslips were washed once with PBS at room temperature and then fixed in 0.2% of glutaraldehyde in PBS for 5 min. The coverslips were washed three times with PBS for 5 min each time at room temperature. Finally, the coverslips were stained for SA-β-galactosidase activity for 24 h at 37 °C in a buffer containing 0.1 mg/mL X-gal, 150 mM NaCl, 2 mM MgCl_2_, 5 mM K_3_Fe(CN)_6_, 5 mM K_4_Fe(CN)_6_, and 40 mM sodium phosphate at pH 6.0. Images were acquired under a tissue culture microscope (Lecia DMi1, Leica Microsystems, Wetzlar, Germany).

### 4.6. DNA Content Analysis with Flow Cytometry

Cells were fixed in 75% ethanol, resuspended in the PBS buffer containing 100 μg/mL of RNase and 10 μg/mL of propidium iodide (Sigma–Aldrich, St. Louis, MI, USA), and then analyzed on a FACS Calibur flow cytometer using CellQuestPro Software (BD Biosciences, San Jose, CA, USA). Cells with 2N or 4N DNA content were quantified by using the FlowJo software (Tree Star, Ashland, OR, USA).

### 4.7. Soft Agar Assay

As described before [45], the soft agar assay was performed in 6-well plates with the bottom layer consisting of 0.7% agar in DMEM. Approximately 10^4^ cells were used for each well. Cells were suspended in 0.35% agar in the growth medium to form the upper layer, which was allowed to solidify at room temperature for 30 min. The upper layer was then covered with 100 μL of growth medium. Cells were incubated at 37 °C in the presence of 5% CO_2_. To each well, 100 μL of DMEM was added twice weekly to prevent the desiccation of agar until colonies became evident. RPE-MYC cells engineered to overexpress MYC were transformed and used as a positive control.

### 4.8. In Vivo Tumorigenicity Assay (Mouse Xenograft Models)

#### 4.8.1. Mouse Information

Eight-week-old female immunocompromised Nu/Nu mice (Chengdu Dossy Experimental Animal Co., Ltd., Chengdu, China) were used for the in vivo tumorigenicity assay study.

#### 4.8.2. Experimental Phase

Animals were allowed to acclimate for at least one week before being randomized into two groups for the implantation experiment. Randomization was performed by using randomize R (version 2.0.0) in R (version 4.1.2) R Foundation, Vienna, Austria [46]. Approximately one million tetraploid RPE-NEO cells in 100 μL of PBS were subcutaneously injected into a dorsal flank of an 8-week-old female immunocompromised Nu/Nu mouse (Chengdu Dossy Experimental Animal Co., Ltd., Chengdu, China). Both flanks of each mouse were implanted. RPE-MYC cells were used as the positive control. The implanted mice were observed for 6–8 months for tumor formation before being euthanized. During the study, mice were euthanized by CO_2_ asphyxiation followed by cervical dislocation. The CO₂ flow rate displaced 30% to 70% of the cage volume per minute. The tumor incidents were counted.

### 4.9. Statistical Analysis

Statistical analyses were performed with R software (version 4.1.2, R Foundation, Vienna, Austria). A two-sided non-paired t-test was used for two-group comparisons to test the null hypothesis that the difference in group means is zero. Multiple group comparisons using t-tests will inflate the probability of declaring a significant difference when it is not present. We, therefore, used two-step procedures for more than two-group comparisons. First, F statistics from one-way ANOVA were computed using the aov function from the stats library (version 4.1.2, R Foundation, Vienna, Austria). The F statistic tests the null hypothesis that all group means are equal. The F statistic was performed at a 5% significance level. Second, when the null hypothesis of the F-test was rejected, either a Dunnett’s test was run to compare multiple groups to a given group or a Tukey test was run to compare the group means between all groups. Dunnett’s two-sided tests were computed using the Dunnett Test function from the DescTools library (version 0.99.44, R Foundation, Vienna, Austria). Tukey tests were computed using the Tukey HSD function from the stats library (version 4.1.2, R Foundation, Vienna, Austria). The different statistical tests used for individual experiments are described in the figure legends. Results were deemed significant if *p* < 0.05 and were denoted as *, *p* < 0.05.

## Data Availability

All data analyzed during this study are included within the manuscript (and its Supplementary Information Files). Data deposition does not apply to the current study.

## Supplementary Materials

The online version contains supplementary material. The following supporting information can be downloaded at: https://www.mdpi.com/article/10.3390/ijms232012104/s1.
