# Supplementary material for: The Effect of Circumscribed Exposure to the Pan-Aurora Kinase Inhibitor VX-680 on Proliferating Euploid Cells
Source: Int J Mol Sci. 2022 Oct 11;23(20):12104. doi: 10.3390/ijms232012104 (PMC9603438; doi:10.3390/ijms232012104)
Supplement: Supplementary file 1 [file ijms-23-12104-s001.zip › ijms-1919016-supplementary.pdf]

Supplementary Information

The Effect of Circumscribed Exposure to the Pan-Aurora Kinase Inhibitor VX-680 on Proliferating Euploid Cells

Xumei Liu[1], Qiong Shi[2], Namrta Choudhry[2]*, Ting Zhang[2], Hong Liu[3], Shenqiu Zhang[4], Jing Zhang[2]* & Dun Yang[1, 2]

[1] Hospital of Chengdu University of Traditional Chinese Medicine, Chengdu 610075, China;
[2] Chengdu Anticancer Bioscience and J. Michael Bishop Institute of Cancer Research, Chengdu 610000, China;
[3] Anticancer Bioscience (U.S.), San Francisco, California 94080, USA;
[4] Anticancer Bioscience (U.K.), St Andrews KY16 9QD, UK

* Correspondance: nchoudhry@anticancerbio.com (N.C.);
jzhang@anticancerbio.com (J.Z.)

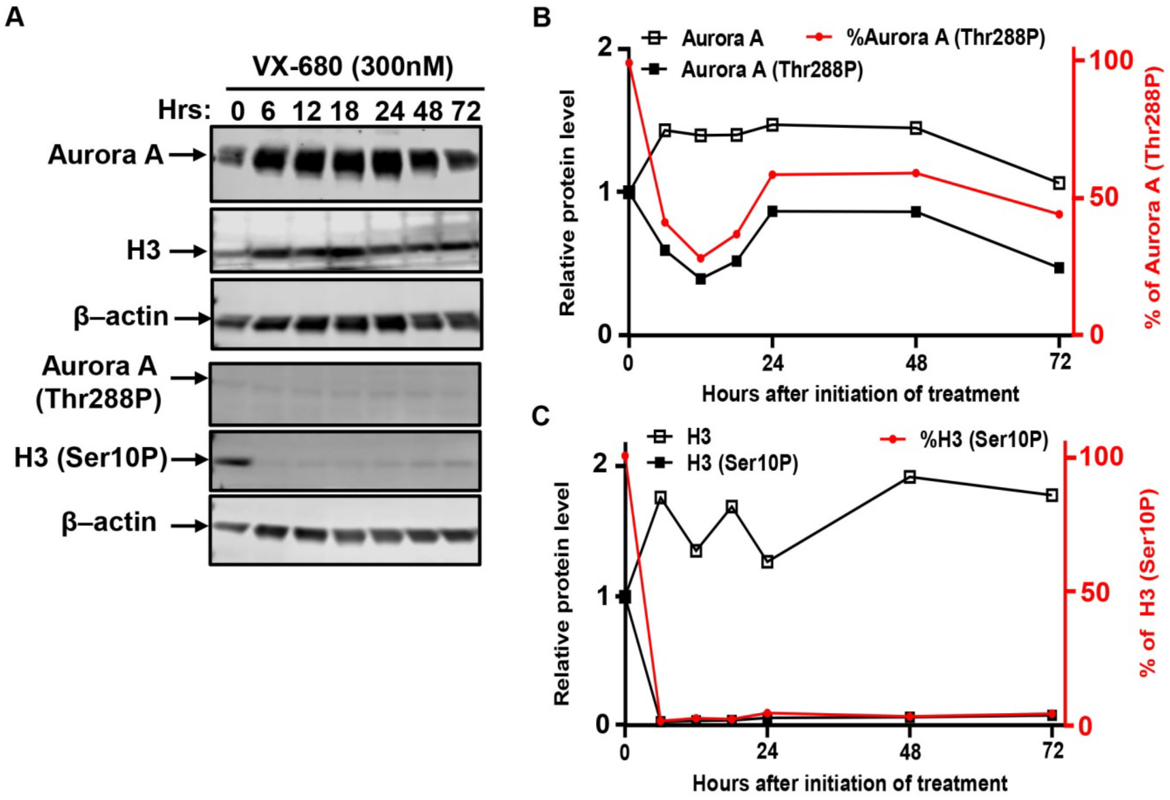

Figure S1. The impact of VX-680 on the Aurora kinases. The blots were cropped and the full-length blots are presented in supplementary figure **S5A-5B.**

RPE-NEO were exposed to 300 nM of VX-680 for three days and then collected at the indicated time points after the initiation of drug treatment. Western blot analysis of the indicated proteins (**A**). Quantification of total AURKA, phosphorylated AURKA at Thr288, and the AURKA kinase activity indicated by its autophosphorylation at Thr288 is presented in (**B**). Quantification of the abundance of total Histone 3, phosphorylated Histone 3 at Ser10, and the percentage of this phosphorylation as a surrogate of the AURKB kinase activity is displayed in (**C**).

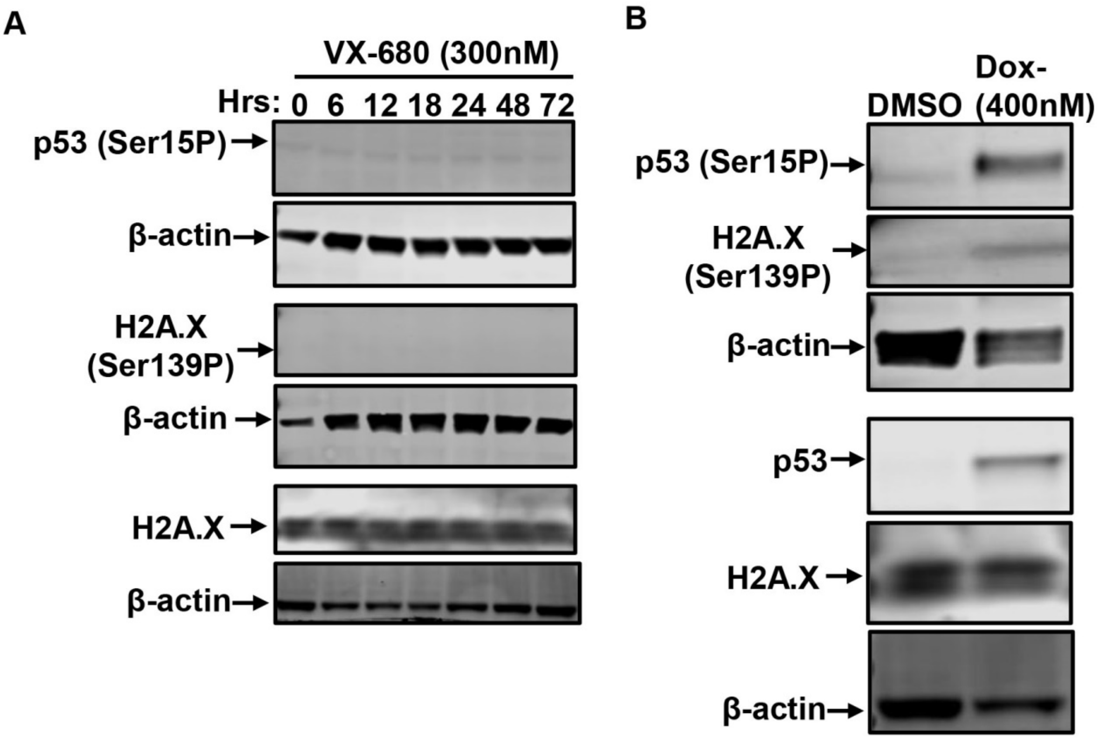

Figure S2. VX-680 fails to elicit the DNA damage response in euploid cells. The blots were cropped and the full-length blots are presented in supplementary figure **S5B-F.**

RPE-NEO cells were exposed to either 300 nM of VX-680 (**A**) or 400 nM of Doxorubicin (**B**) for three days. The cells were collected at the indicated time points after the initiation of drug treatment for analysis of the indicated DNA damage response marker proteins by Western blot.

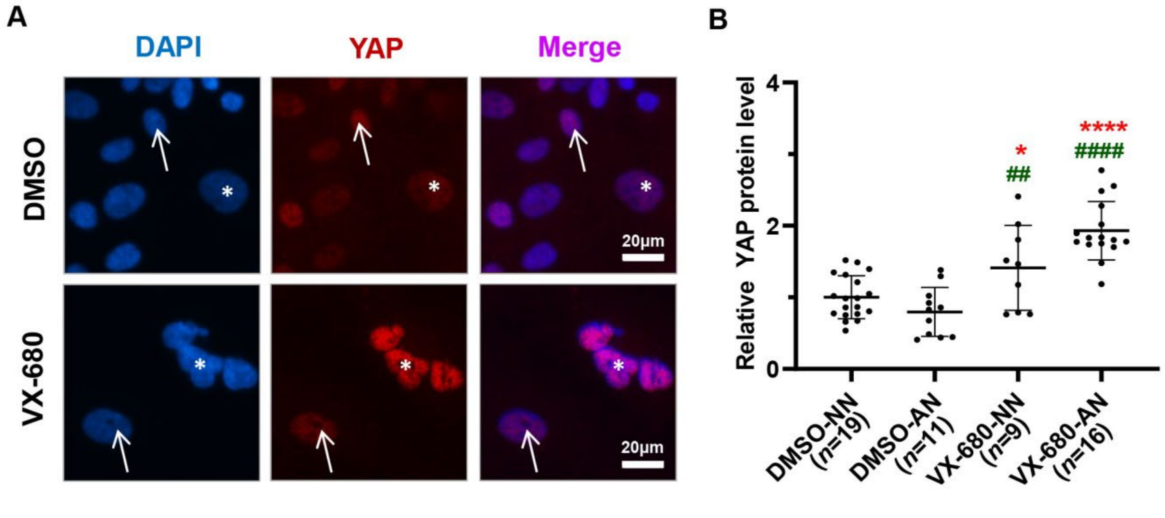

Figure S3. Treatment of euploid cells with VX-680 results in a moderate upregulation of the nuclear YAP.

RPE-NEO cells were treated for 3 days with either 300 nM of VX-680 or DMSO. The treated cells were then analyzed for YAP by immunofluorescence (**A**). Arrow denotes cells with a normal nucleus (NN) and stars point to cells with an abnormal nucleus (AN). Quantification of nuclear YAP in cells with a NN or AN in both the DMSO group and VX-680 group is presented in (**B**). In each experiment, 9-19 nuclei in each group were randomly chosen for quantification of the nuclear YAP signal. The data were normalized to the average value of the DMSO-NN group and a representative experiment is shown. The p values in (**B**) were calculated with the Dunnett test following a one-way ANOVA. *, $p < 0.05$ and ****, $p < 0.0001$, when compared with the DMSO-NN group. ##, $p < 0.01$ and ####, $p < 0.0001$, when compared with the DMSO-AN group.

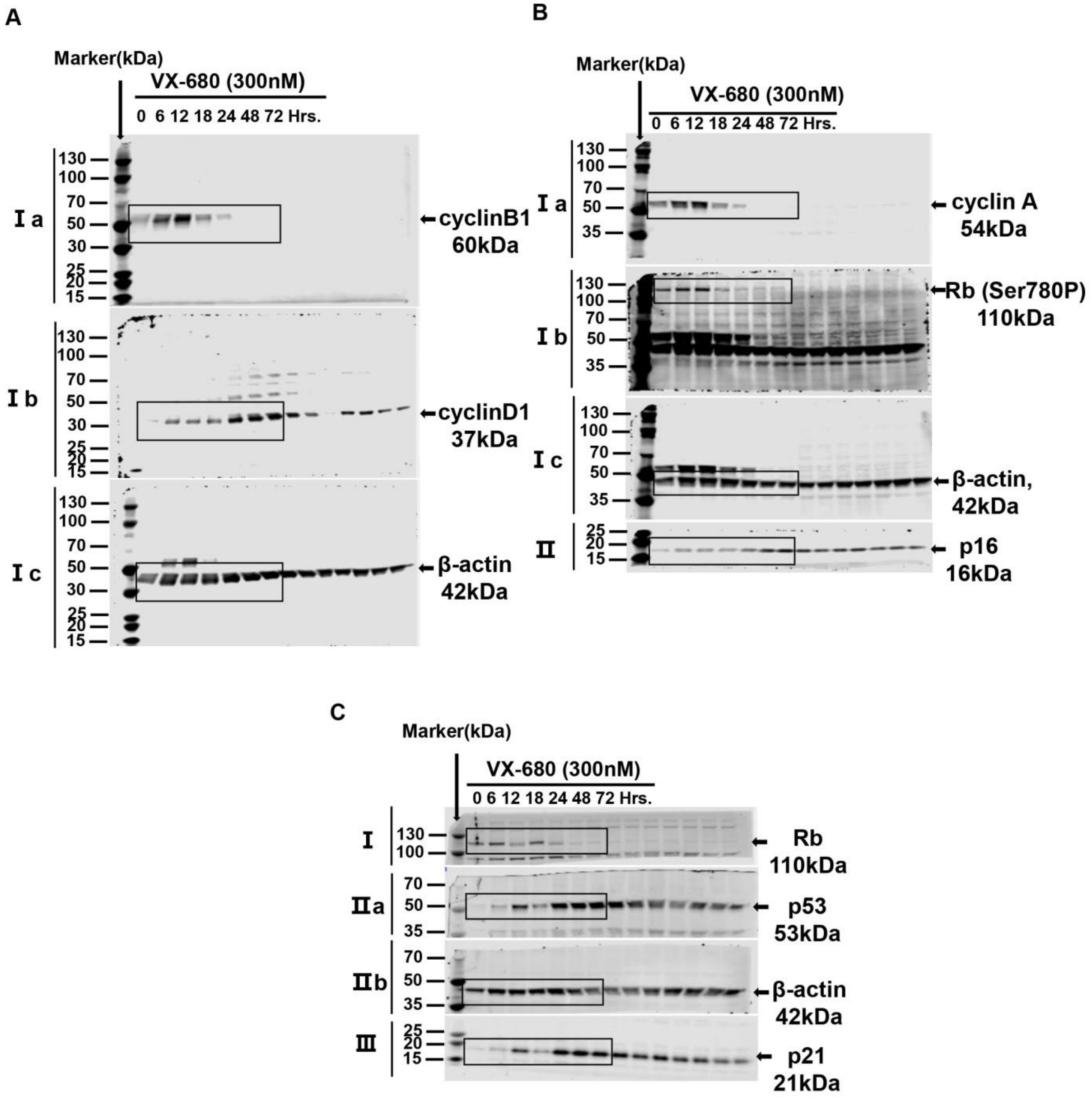

Figure S4. The full-length blots for the main figures **1-2**.

A whole membrane was sequentially probed for cyclin B1 (Ia), cyclin D1 (Ib), and β-actin (Ic) (**A**). One membrane was cut into the upper part (I) and the bottom part (II). The upper part was sequentially probed for cyclin A (Ia), Rb Ser 780P (Ib), and β-actin (Ic). The bottom part was probed for p16 (II) (**B**). One membrane was cut into three parts (I, II & III). Part I and III were analyzed for total Rb and p21, respectively. Part II was sequentially probed for p53 (IIa) and actin (IIb), respectively (**C**).

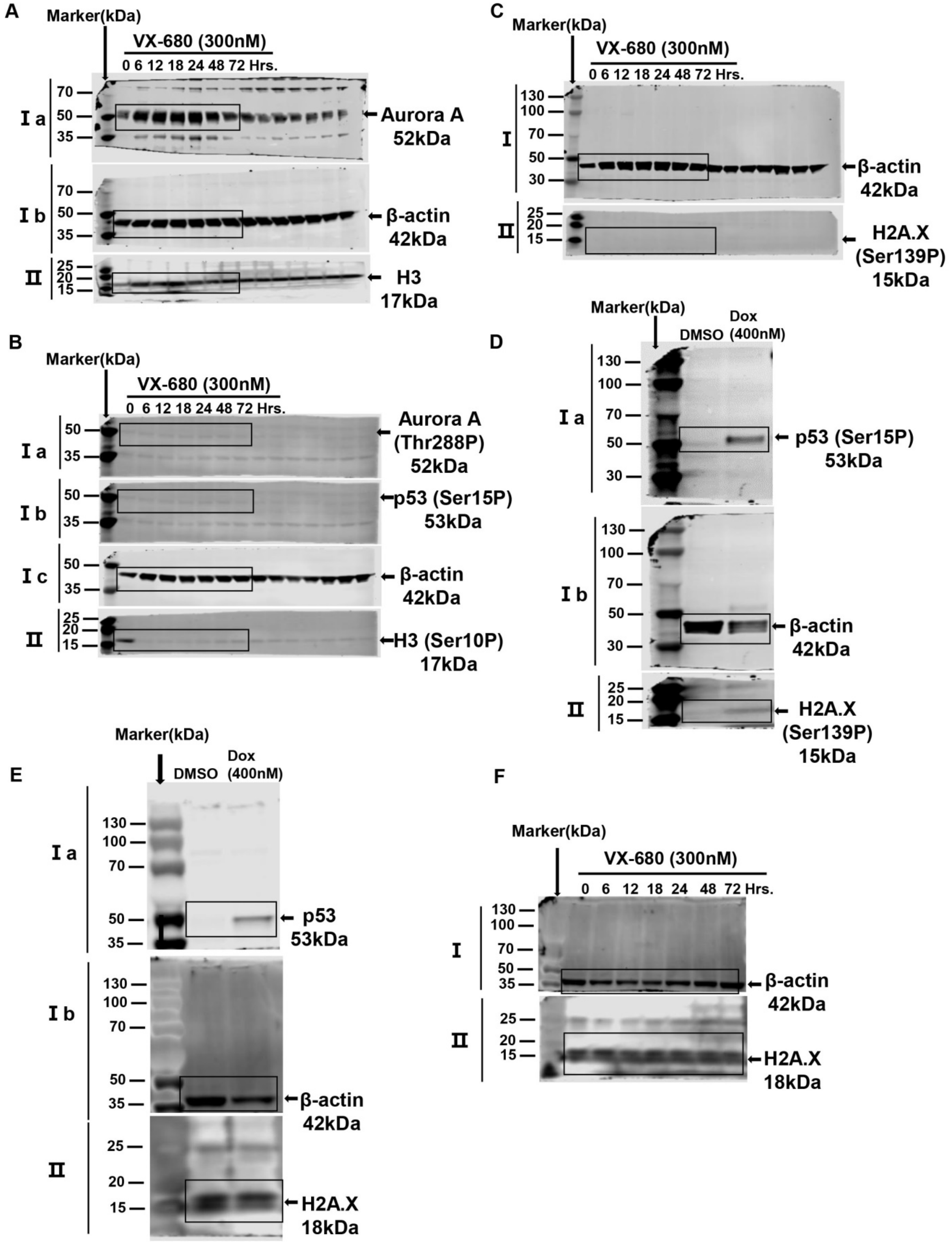

Figure S5. The full-length blots for the supplementary figures **S1-2**.

One membrane was cut into the upper part (I) and the bottom part (II). The upper part was sequentially probed for AURKA (Ia) and β-actin (Ib), respectively. The bottom part was probed for H3 (II) (**A**). One membrane was cut into the upper part (I) and the bottom part (II). The upper part was sequentially probed for AURKA Thr288P (Ia), p53 Ser15P (Ib), and β-actin (Ic), respectively. The bottom part was analyzed for H3Ser10P (II) (**B**). One membrane was cut into two parts which were separately probed for β-actin (I) and H2A.X Ser139P (II), respectively (**C**). One membrane was cut into the upper part (I) and the bottom part (II). The upper part was sequentially probed for p53 Ser15P (Ia) and β-actin (Ib), respectively. The bottom part was analyzed for H2A.X Ser139P (II) (**D**). One membrane was cut into the upper part (I) and the bottom part (II). The upper part was sequentially probed for p53 (Ia), and β-actin (Ib), respectively. The bottom part was analyzed for H2A.X (II) (**E**). One membrane was cut into the upper part (I) and the bottom part (II). The upper part was probed for β-actin (I). The bottom part was analyzed for H2A.X (II) (**F**).
